# Pilot study of the DART tool - an objective healthcare simulation debriefing assessment instrument

**DOI:** 10.1186/s12909-022-03697-w

**Published:** 2022-08-22

**Authors:** Kaushik Baliga, Andrew Coggins, Sandra Warburton, Divya Mathias, Nicole K. Yamada, Janene H. Fuerch, Louis P. Halamek

**Affiliations:** 1 Sydney Medical School, Westmead Hospital, Block K, Level 6, Sydney, NSW 2145 Australia; 2 Simulated Learning Environment for Clinical Training (SiLECT), Westmead Hospital, Sydney, NSW 2145 Australia; 3 The Australian Institute of Medical Simulation and Innovation (AIMSi), Blacktown Hospital, Sydney, NSW 2148 Australia; 4 Department of Pediatrics, Division of Neonatal and Developmental Medicine, Stanford University School of Medicine, Palo Alto, CA USA

**Keywords:** Educational measurement, Feedback, Humans, Reproducibility of results, Simulation training, Staff development

## Abstract

**Background:**

Various rating tools aim to assess simulation debriefing quality, but their use may be limited by complexity and subjectivity. The Debriefing Assessment in Real Time (DART) tool represents an alternative debriefing aid that uses quantitative measures to estimate quality and requires minimal training to use. The DART is uses a cumulative tally of instructor questions (IQ), instructor statements (IS) and trainee responses (TR). Ratios for IQ:IS and TR:[IQ + IS] may estimate the level of debriefer inclusivity and participant engagement.

**Methods:**

Experienced faculty from four geographically disparate university-affiliated simulation centers rated video-based debriefings and a transcript using the DART. The primary endpoint was an assessment of the estimated reliability of the tool. The small sample size confined analysis to descriptive statistics and coefficient of variations (CV%) as an estimate of reliability.

**Results:**

Ratings for Video A (*n* = 7), Video B (*n* = 6), and Transcript A (*n* = 6) demonstrated mean CV% for IQ (27.8%), IS (39.5%), TR (34.8%), IQ:IS (40.8%), and TR:[IQ + IS] (28.0%). Higher CV% observed in IS and TR may be attributable to rater characterizations of longer contributions as either lumped or split. Lower variances in IQ and TR:[IQ + IS] suggest overall consistency regardless of scores being lumped or split.

**Conclusion:**

The DART tool appears to be reliable for the recording of data which may be useful for informing feedback to debriefers. Future studies should assess reliability in a wider pool of debriefings and examine potential uses in faculty development.

## Background

Simulation-based medical education (SBME) allows participants to safely apply skills in a team-based context with debriefing allowing for collective reflection and learning [1, 2]. Facilitation of debriefings is viewed as a difficult skill to master. Effective debriefers are often seen to encourage reflection, uncover performance gaps and promote a discussion of how to improve management of future scenarios [3, 4].

Debriefing is recognized as an essential component of SBME delivery [3]. Assessments of debriefing quality assist in improving the future performance of debriefers [5]. A number of recognized scoring aids are commonly used to assess debriefing quality including the Objective Structured Assessment of Debriefing (OSAD), and the Debriefing Assessment for Simulation in Healthcare (DASH) tools [2, 6]. These aids assess debriefers’ performance on a Likert-scale based on specific observable behaviors [1, 6]. For instance, in the DASH debriefers are assessed globally on their ability to provide an “engaging learning environment” and explore “performance gaps” [1]. These tools provide a useful framework and are demonstrative of ideal behaviors but are not without limitations. First, they are relatively time consuming and use subjective scales. For instance, what may be considered an engaging learning environment for one rater may be viewed as challenging, onerous, or problematic by other raters. Local culture is widely understood to influence engagement and expectations during debriefings and therefore may undermine the accuracy of the various tools [7, 8]. Furthermore, similar survey tools may lead to response biases in raters [9]. These biases could diminish the reliability of Likert-scale scoring of debriefing assessment tools. We have observed this as provision of socially desirable (higher) ratings in a peer context or extreme responding (e.g., blanket scoring of 7/7 in all domains) [10, 11]. To summarize, despite widespread use of SBME for healthcare professions learning, our current assessment tools for debriefer performance are qualitative, subjective, and focus only on ideal behaviors. Therefore, a gap exists for complementary ‘quantitative’ approaches to rating performance and providing feedback. To address this issue, we propose a new scoring system - ‘The Debriefing Assessment in Real Time (DART) tool’. The goal of this pilot study was to explore and investigate the reliability and potential utility of the DART tool as an alternative approach to assessment of debriefing quality.

## Methods

### Study setting

This international study was a collaboration between the Center for Advanced Pediatric and Perinatal Education (CAPE) at Stanford University (USA) and three Australian hospitals affiliated SBME centers in Western Sydney. A supervising author (LPH) has over 25 years of SBME experience and conceived the Debriefing Assessment in Real Time (DART) tool following observation of simulation and debriefing at the National Aeronautics and Space Administration (NASA) and extensive debriefing experience with CAPE faculty [12]. As stated above the stated goals were to explore and investigate the reliability and potential utility of the DART tool as an alternative approach to assessment of debriefing quality.

### DART tool

**Fig. 1:**
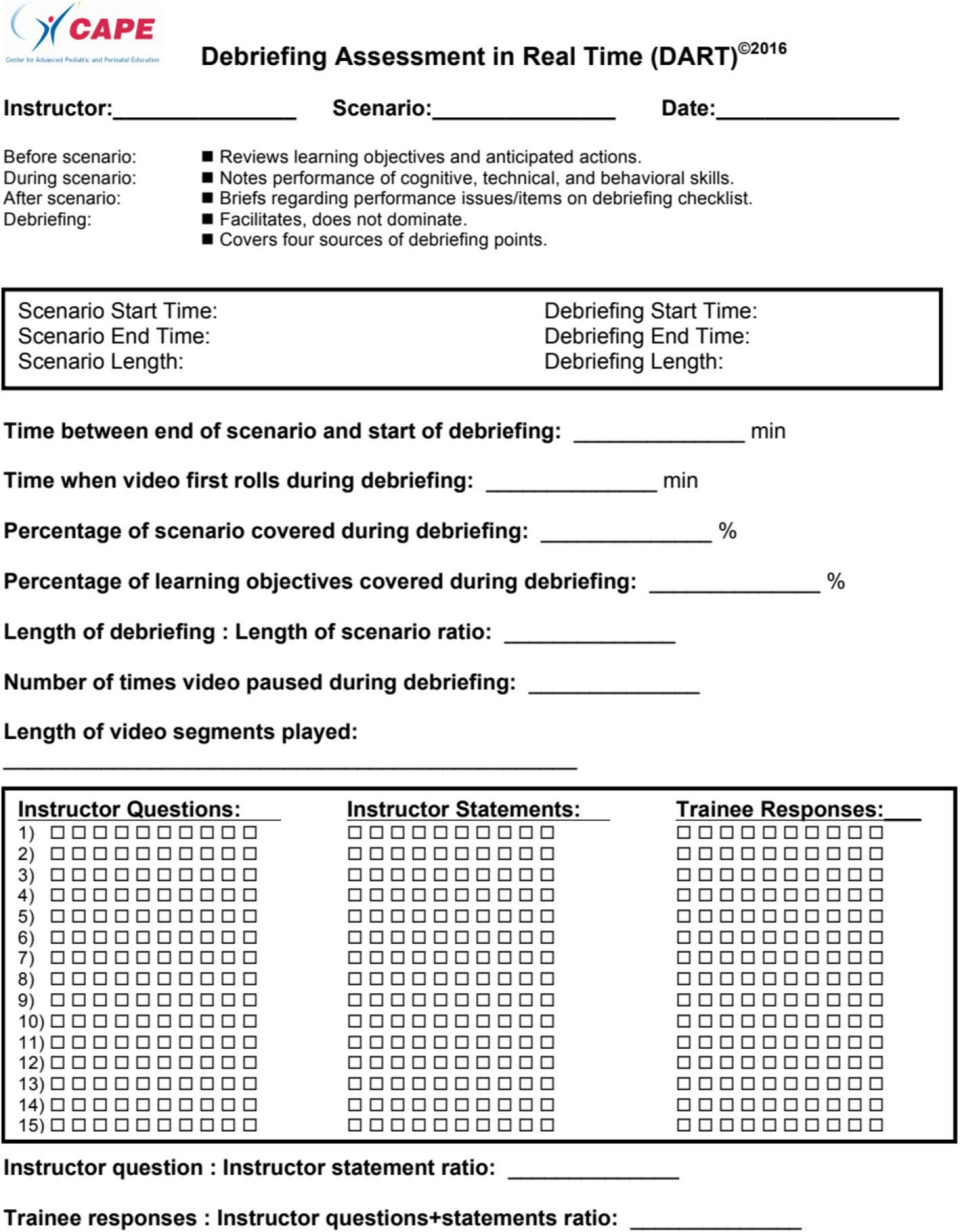
CAPE Debriefing Assessment in Real Time (DART) Tool

The DART (Fig. 1) was developed as a real-time objective measure of debriefing performance by faculty at the Center for Advanced Pediatric and Perinatal Education (CAPE) based on practices in simulation and debriefing in non-healthcare industries. This tool scores observable sequential debriefing contributions in a cumulative fashion including Instructor Questions (IQ), Instructor Statements (IS) and Trainee Responses (TR). Furthermore, the tool provides information to SBME supervisors on key timings and ratios of instructor questions:statements (IQ:IS) and trainee:instructor verbalizations (TR:[IQ + IS]) can be calculated.

### Subject selection

Eligible subjects were interdisciplinary adult simulation faculty with a formal simulation center or university affiliation. No specific exclusion criteria were determined prior to subject selection as this was an explorative project for the generalizability of the DART tool. Subjects were faculty who volunteered their time for the pilot study.

### Study overview

Two pre-filmed video examples (Video A and Video B) of post-simulation debriefing were selected for the assessment of the DART. Using the DART, subjects (*n* = 8) individually rated the debriefings while watching the video. Printed paper copies of the DART tool (Fig. 1) were used to score Video A and Video B in real time (in a single take) as per instructions of the tool’s designer (LPH). Videos were viewed separately on desktop computers to ensure subjects were blinded to each other’s scores. Responses were collated and tabulated by a single investigator (KB).

#### Video transcript

**Fig. 2:**
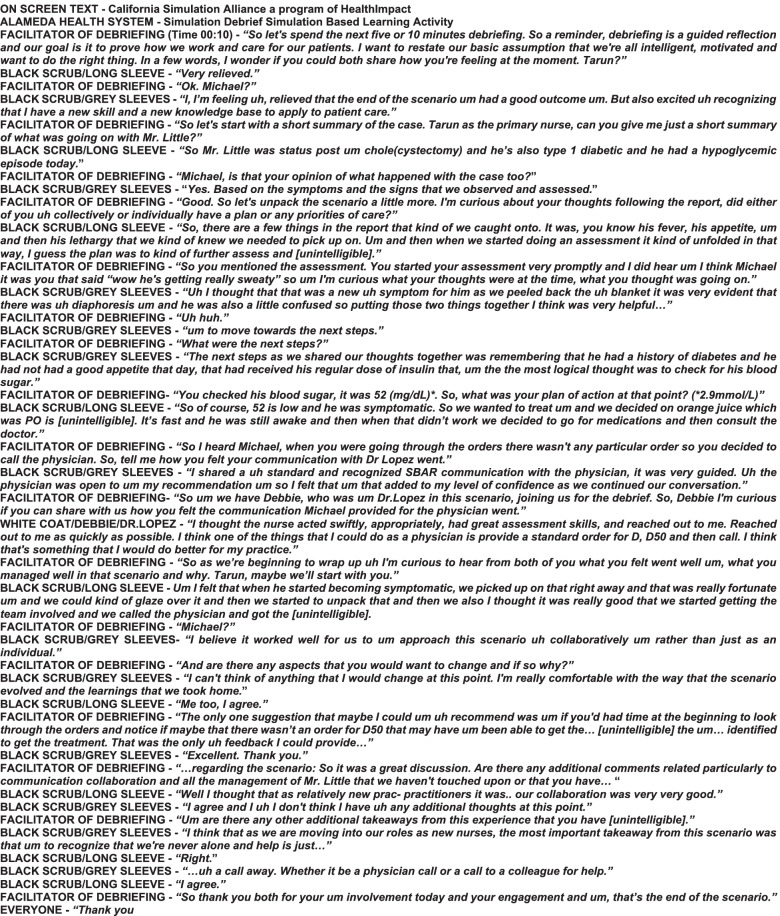
Video A Transcript

Video A was selected for additional assessment. A university staff member with training in qualitative methods professionally transcribed the video (Fig. 2). The use of a transcript for rating was intended to provide an in-depth analysis identifying areas where subjects differed the most in their recorded observations. Subjects were instructed to highlight sentences that translated to their recorded observations while they rated the transcript using the DART. In order to ensure accuracy, subjects were not limited to only reading sentences once. Upon completion, a discussion took place among subjects regarding their reasoning behind their DART scores.

#### Calibration video selection

The two short debriefing videos were selected from Free Open Access Medical Education sources. Sample brief debriefing videos (purporting to represent good performance) from various formal simulation organizations were reviewed and after consultation among our collaborative research group two contemporary videos were selected for this pilot study. Video A (California Simulation Alliance) exemplified a predominance for an Advocacy-Inquiry approach to debriefing, whereas Video B (The Patient Safety Institute) exemplified the D.E.B.R.I.E.F. model of debriefing:

- Debriefing Video A (December 2018) - California Simulation Alliance Health Impact (Origin – United States). Description - 'Filmed on location at Highland Hospital in Oakland, California' [13].
- Debriefing Video B (March 2016) - The Patient Safety Institute (Origin – United States) - Description - 'This demonstrates what a good debrief looks like using the D.E.B.R.I.E.F. method' [14].

### Analysis

As per the DART, subjects recorded the number of instructor questions (IQ), instructor statements (IS), and trainee responses (TR). Two different ratios were calculated from the recorded values: A ratio of instructor questions to instructor statements (IQ:IS) and a ratio of trainee responses to instructor questions and statements (TR:[IQ + IS]). In this study, inter-rater reliability of the DART was estimated by a calculated Coefficient of Variation (CV%). The CV% describes the dispersion of data relative to its mean. CV% for each reported cumulative tally and ratio were calculated using descriptive statistics (SD ÷ mean). CV% was selected for statistical analysis rather than Intraclass Correlation Coefficients (ICC) because of the limited sample size. It is recommended to include 30 or more samples involving at least 3 raters in order to interpret the ICC accurately [15].

Values of CV% were compared within each of the three data debriefings (Video A, Video B, and Transcript A) in order to estimate variability in ratings. We compared the mean CV% of the recorded observations to each calculated ratio within each data set. Additionally, we compared the CV% between the ratings for videos versus those for the transcript. Finally, we compared the mean CV% of each individual recorded observation and calculated the ratio between each data set.

## Results

Tables 1, 2 and 3 show each subject’s (*n* = 8) demographic characteristics, self-reported DART scores and calculated ratios for Video A (*n* = 7), Video B (*n* = 6), and the transcript of Video A (*n* = 6). Due to limited availability, not all subjects were able to rate each video and transcript. Subjects used were experienced in simulation and debriefing, with a median of 9.0 (IQR 7.5-12.5) years of experience. There were more subjects with a physician background (*n* = 5) than a nursing background (*n* = 3).

**Table 1:** Video A

Rater	Format	Role	Rater Exp. (years)	Rater Sex (m/f/o)	Instructor Questions (IQ)	Instructor Statements (IS)	Trainee Responses (TR)	IQ:IS Ratio	TR:[IQ + IS] Ratio
1	video	MD	21	M	8	22	37	0.36	1.23
2	video	MD	10	F	13	11	19	1.18	0.79
3	video	MD	15	F	6	19	11	0.32	0.44
4	video	RN	6	F	13	12	24	1.08	0.96
5	video	MD	9	M	11	10	15	1.10	0.71
6	video	MD	3	M	11	11	25	1.00	1.14
7	video	RN	9	F	15	13	20	1.15	0.71
Sum			73		77.0	98.0	151.0	6.20	5.99
Mean			10.4		11.0	14.0	21.6	0.89	0.86
SD			5.94		3.1	4.6	8.4	0.38	0.27
CV%			–		28.3%	33.0%	38.8%	42.6%	32.0%

Global Impression of Debriefing Quality: ‘The facilitator leads, appears inclusive and uses “Advocacy with Inquiry”. Reflection is encouraged through use of a series of effective questions. Performance gaps are not addressed in detail. All participants contribute to the conversation’

**Table 2:** Video B

Rater	Format	Role	Rater Exp. (years)	Rater Sex (m/f/o)	Instructor Questions (IQ)	Instructor Statements (IS)	Trainee Responses (TR)	IQ:IS Ratio	TR:[IQ + IS] Ratio
1	video	MD	21	M	6	37	22	0.16	0.51
2	video	MD	10	F	6	13	19	0.46	1.00
3	video	MD	15	F	12	14	19	0.86	0.73
4	video	RN	6	F	8	12	10	0.67	0.50
5	video	MD	9	M	7	16	11	0.44	0.48
6	video	MD	3	M	5	12	11	0.42	0.65
Sum			64		44.0	104.0	92.0	3.00	3.87
Mean			10.7		7.3	17.3	15.5	0.50	0.64
SD			6.47		2.5	9.8	5.3	0.24	0.20
CV%			–		34.1%	56.3%	34.2%	47.5%	31.0%

Global Impression of Debriefing Quality: ‘Facilitator(s) lead strongly and appear to dominate the debriefing to the point of providing mini-lectures rather than facilitating reflection. Performance gaps were briefly addressed’

**Table 3:** Transcript for Video A

Rater	Format	Role	Rater Exp. (years)	Rater Sex (m/f/o)	Instructor Questions (IQ)	Instructor Statements (IS)	Trainee Responses (TR)	IQ:IS Ratio	TR:[IQ + IS] Ratio
1	transcript	MD	21	M	11	27	43	0.34	1.13
2	transcript	MD	10	F	10	14	21	0.71	0.88
3	transcript	MD	9	M	16	14	32	1.14	1.07
4	transcript	MD	3	M	17	17	51	1.00	1.50
5	transcript	MD	9	F	16	18	34	0.89	1.00
6	transcript	RN	9	F	16	14	27	1.14	0.90
Sum			61		86.0	104.0	208.0	5.30	6.47
Mean			10.2		14.3	17.3	34.7	0.88	1.08
SD			5.88		3.0	5.0	10.9	0.28	0.23
CV%			–		21.0%	29.1%	31.3%	32.2%	21.1%

Global Impression of Debriefing Quality: ‘The facilitator leads, appears inclusive and uses “Advocacy with Inquiry”. Reflection is encouraged through use of a series of effective questions. Performance gaps are not addressed in detail. All participants contribute to the conversation’

The mean of each individual variable across all three data sets (Video A, Video B, and the Transcript), and the mean of all reported observations (IQ, IS, and TR) within a data set were calculated for analysis. We found the mean CV% for the three reported observations in Video A, Video B, and the transcript was 33.3, 41.5, and 27.1%, respectively. When comparing these values with the CV% values of both ratios, we found them lower for TR:[IQ + IS], but higher for IQ:IS. Further, we found the CV% for each variable in the transcript (IQ = 21.0%, IS = 29.1%, TR = 31.3%, IQ:IS = 32.2%, TR:[IQ + IS] = 21.1%) lower than the same CV% values for Video A (IQ = 28.3%, IS = 33.0%, TR = 38.8%, IQ:IS = 42.6%, TR:[IQ + IS] = 32.0%) and Video B (IQ = 34.1%, IS = 56.3%, TR = 34.2%, IQ:IS = 47.5%, TR:[IQ + IS] = 31.0%). When comparing individual scores across each data set, the mean CV% for IQ (27.8%) was lower than the mean CV% for IS (39.5%) and for TR (34.8%). Additionally, the mean CV% for IQ:IS ratio (40.8%) was higher than the mean CV% for either of the individual scores used in the ratio (IQ = 27.1%, IS = 39.5%).

## Discussion

In this study we explored the use of DART as a simple and objective scoring system for recorded interdisciplinary healthcare simulation debriefings. We assessed heterogenous sources (debriefings and transcripts) and enrolled eight interdisciplinary raters from four simulation centers to estimate variation in scoring. Observed variances in IQ, IS, TR and IQ:IS were higher compared with the TR:[IQ + IS] ratio. The difference may be attributable to whether raters were “lumpers” or “splitters” in their characterization of long statements as single or multiple concepts. “Lumpers” are study subjects who had the tendency to score long statements as a single concept, and “splitters” as subjects who had the tendency to score the longer statements as multiple concepts. Regardless of whether subjects were considered “lumpers” or “splitters”, the low variance in TR:[IQ + IS] suggests the DART is internally consistent. Furthermore, we observed a lower mean variance for IQ in comparison to IS or TR (Tables 1, 2 and 3). The lower variance in identification of questions (IQ) indicates that debriefing raters are readily able to recognize questions compared to statements. We note that with the commonly used advocacy-inquiry (AI) approach to debriefing, the debriefers often mix statements and questions together. This in turn could reduce reliability of DART scores as well as the inferences drawn about quality from the tool. For example, a debriefer using AI may make more statements and ask less questions and therefore, from their DART score, appear less effective or less inclusive facilitator. Of course, the opposite may be true. While we recognize this as a limitation of the DART tool for measuring debriefing quality, the tool scores could still be used as a basis for giving peer-feedback to debriefer colleagues. For instance, one might share with a colleague: *“I noticed that you asked 3 questions and made 25 statements about respiratory failure. This count back of your questions and statements might suggest some room for improvement in our encouragement of reflection in this debriefing. What was your thought process at the time?”* Furthermore, in reflecting on why the alternative debriefing assessment tools like the DART is likely to useful in many simulation settings we ask the reader to consider if they ever or often observe either a lack of questions or lecturing by debriefers? [7] We do not by any means claim these behaviors are universal, a predominance of the debriefer talking has been a proven observation across the simulation sites where this study was based [16].

When comparing the scores of videos as compared to the transcript, we found lower variances for each reported observation and calculated ratio in the transcript scores. Subjects rating the transcript had no limitations regarding rereading sentences, while subjects rating Videos A and B were unable to rewind or rewatch film and were limited to watching in real time. While accuracy increases with the ability to reread and reflect, these circumstances do not represent practical use of the DART. As a result, transcript scores may underestimate the true variation in scoring, and videos A and B may better represent the real-world use of DART [17]. It may be easier to determine the breakdown of statements in the scoring of a written transcript but use in real-time leading to variation in scoring is unlikely to preclude the tool’s usefulness in faculty feedback. Moreover, it is recognized in high stakes assessments that observer error is a significant problem [18, 19]. Similarly, debriefing scoring variation could be prone to rater error. However, given the intended use of the DART in debriefing for new faculty feedback, the thresholds of acceptable error may be wider than for high-stakes assessments. As a result, in our view the CV% observed in this study are acceptable for further work that tests the validity of the tool for faculty development. One issue that has not been clarified at this point is what various DART ratios scores represent in terms of a representation of true debriefing quality. Sanders’ prior work on promoting reflective practice may suggest that higher cumulative tallies of questions (IQ) and participant contributions (TR) are observed in debriefings where reflection and practice change is being promoted [20].

In terms of specific problems with the DART tool, we identified more errors in IS scores. After discussion with each rater and review of our transcript we believe this variation may be attributable to a “lumper/splitter” phenomenon. Variation in each rater’s assessment of a single “statement” or “single concept” appears to be problematic and may have led to the higher CV% observed for IS. As an example, we can address this statement taken from the transcript: *“So let’s spend the next five- or 10-minutes debriefing. So, a reminder, debriefing is a guided reflection, and our goal is to improve how we work and care for our patients. I want to restate our basic assumption that we’re all intelligent, motivated and want to do the right thing*” When asked about their scores, raters that were “lumpers” may have considered this as a single statement, giving a score of one. However, “splitters” may consider each sentence in the quote as a separate statement, giving a score of three. Implementing a standardized training protocol and calibration exercises may reduce these differences, but it is not our intention to increase cognitive load or over complicate the use of a tool that was designed to be easy to use [21].

### Comparison with other rating tools

The SBME literature outlines a range of ideal behaviors exhibited by debriefers that can promote reflective practice and improve performance [3]. Existing models of providing feedback have a key role in identifying the factors listed but may fail to provide quantitative information to debriefers seeking to understand performance. Further, existing tools (i.e., DASH and OSAD) have notable limitations in their validation studies and may be subject to “response bias”, which is a problem with Likert scales [9].

The OSAD tool [6], which has recently been validated electronically and in languages other than English, provides useful feedback to debriefers, but also uses a relatively subjective 1-5 Likert rating scale [22, 23]. Further, while the OSAD tool has been studied for a wider range of settings than the DASH tool, including pediatric simulation, one of the major validation studies used just two raters to examine the tool [6, 23]. Of note, a recently described tool known as the Simulation in Healthcare retrOaction Rating Tool (SHORT) was described as an alternative approach for shorter debriefings [24]. The authors simultaneously derived and validated their tool, which appears to have excellent agreement and good inter-rater reliability when used for assessing SBME.

The widely used DASH tool was validated with the use of 3 debriefing example videos that were scored by more than 100 online raters [1]. However, the DASH has neither been externally validated nor translated into other languages or formats. In addition, from a user standpoint, it is challenging to use the DASH tool for feedback after the debriefing because the attributed scores do not provide specific goals to target in the next debriefing opportunity in terms of definitive actions or targets. We suggest that the DASH, SHORT or OSAD (which highlight many of the subjective qualities expected of facilitators) could be used in combination with the DART tool to enhance feedback for novice debriefers or for peer coaching of experienced debriefers [25].

### Implications for faculty development

A recent study recognized that traditional methods of SBME faculty development lack a structured approach to achieve expertise and proposed the use of DebriefLiveⓇ, a virtual teaching environment that allows faculty to review their debriefing performances by observing recorded videos and scoring themselves [26]. Direct observation of debriefers by experienced faculty, faculty mentoring to achieve debriefing expertise, and targeted coaching conversations using an agreed-upon approach may all have some role in assisting with the development of skill in debriefing [5, 25, 27]. Moreover, the use of quantitative scoring systems have the potential to provide conversational substrate for all of these approaches, and may help debriefers improve at all levels of experience.

In non-healthcare settings, it is generally established that those participating in debriefings engage with each other in problem solving and the debriefer is generally a *“guide on the side”* rather than *“a sage on the stage”* [3]. However, our personal observation in healthcare simulation practice is that debriefers are frequently in the latter category. In the second video example (Video B) assessed in this study the video publishers cited the video as a “good example” but the debriefer(s) talked for > 80% of the debriefing [14]. We have observed that the length of time talking seems to be a germane factor when assessing the quality of facilitation [16]. Using the OSAD tool or the DASH score for this video debriefing, or a similar real life equivalent, may not have resulted in a true understanding of the issues requiring improvement (i.e. the debriefer dominating the conversation). To summarize, using quantitative data may help amplify feedback to debriefer colleagues and this in turn may help behavior change. The DART tool provides point of care information to debriefers, and this can either supplement the use of the OSAD, DASH or SHORT tools, or be used as a standalone matrix for debriefer feedback. The DART addresses the limitations of qualitative measures by replacing subjective scales with a cumulative scoring method, avoiding response bias, and reducing complexity. This ease of use permits the DART with the potential to track debriefer progression over time by continually comparing current scores to previous ones. From the results of this pilot study, we plan to further assess the reliability and validity of the DART tool by expanding the number of study sites, videos and raters in a future study.

### Limitations

We acknowledge the limitations of our study. Firstly, the limited number of debriefings assessed restricted the quantity of ratings available for analysis. Unlike CV%, which was used in this study and may be a sub-optimal analysis, the ICC would have provided a standardized stratification system for evaluating variability [15]. Secondly, we recognize the creators of this tool are listed as authors which could have led to unrecognized implicit bias in the study. Thirdly, as discussed above, there was no standardized tool orientation used in this study. This may have led to the higher variance in some reported observations. From the experiences of conducting the studies we have made a training video and calibration exercise hosted at www.emergencypedia.com/CAPE which is free to use. Finally, in terms of real-world extrapolation, the DART is meant to be used to evaluate debriefers in real time. However, in this study we used video debriefings and written transcripts which may not represent real world use of the tool.

## Conclusions

The DART tool has the potential to provide reliable data about healthcare simulation debriefing. As a real-time instrument, DART can be used either alone or in conjunction with qualitative tools such as DASH or SHORT for assessing the quality of debriefings [28]. Further evaluation using a spectrum of debriefings at users should now be conducted to determine the best future role of this tool.

## Data Availability

All data generated or analysed during this study are included in this published article**.** SiLECT centre data is available on request from andrew.coggins@health.nsw.gov.au

## Abbreviations

AI: Advocacy-Inquiry
AIMSi: Australian Institute of Medical Simulation and Innovation
CAPE: Center for Advanced Pediatric and Perinatal Education
CV%: Coefficient of Variation
DART: Debriefing Assessment in Real Time
DASH: Debriefing Assessment for Simulation in Healthcare
HREC: Human research and ethics committee
ICC: Intraclass Correlation Coefficient
IQ: Instructor Questions
IS: Instructor Statements
NASA: National Aeronautics and Space Administration
OSAD: Objective Structured Assessment of Debriefing
SBME: Simulation-based medical education
SHORT: Simulation in Healthcare retrOaction Rating Tool
SiLECT: Simulated Learning Environment for Clinical Training
TR: Trainee Responses
WSLHD: Western Sydney Local Health District
